# Physiological implications of arginine metabolism in plants

**DOI:** 10.3389/fpls.2015.00534

**Published:** 2015-07-30

**Authors:** Gudrun Winter, Christopher D. Todd, Maurizio Trovato, Giuseppe Forlani, Dietmar Funck

**Affiliations:** ^1^Laboratory of Plant Physiology and Biochemistry, Department of Biology, University of Konstanz, Konstanz, Germany; ^2^Department of Biology, University of Saskatchewan, Saskatoon, SK, Canada; ^3^Department of Biology and Biotechnology, Sapienza University of Rome, Rome, Italy; ^4^Laboratory of Plant Physiology and Biochemistry, Department of Life Science and Biotechnology, University of Ferrara, Ferrara, Italy

**Keywords:** arginine, arginine biosynthesis, arginase, ornithine aminotransferase, urease, polyamines, nitric oxide

## Abstract

Nitrogen is a limiting resource for plant growth in most terrestrial habitats since large amounts of nitrogen are needed to synthesize nucleic acids and proteins. Among the 21 proteinogenic amino acids, arginine has the highest nitrogen to carbon ratio, which makes it especially suitable as a storage form of organic nitrogen. Synthesis in chloroplasts *via* ornithine is apparently the only operational pathway to provide arginine in plants, and the rate of arginine synthesis is tightly regulated by various feedback mechanisms in accordance with the overall nutritional status. While several steps of arginine biosynthesis still remain poorly characterized in plants, much wider attention has been paid to inter- and intracellular arginine transport as well as arginine-derived metabolites. A role of arginine as alternative source besides glutamate for proline biosynthesis is still discussed controversially and may be prevented by differential subcellular localization of enzymes. Apparently, arginine is a precursor for nitric oxide (NO), although the molecular mechanism of NO production from arginine remains unclear in higher plants. In contrast, conversion of arginine to polyamines is well documented, and in several plant species also ornithine can serve as a precursor for polyamines. Both NO and polyamines play crucial roles in regulating developmental processes as well as responses to biotic and abiotic stress. It is thus conceivable that arginine catabolism serves on the one hand to mobilize nitrogen storages, while on the other hand it may be used to fine-tune development and defense mechanisms against stress. This review summarizes the recent advances in our knowledge about arginine metabolism, with a special focus on the model plant *Arabidopsis thaliana*, and pinpoints still unresolved critical questions.

## Introduction

Plant growth is often limited by the availability of nutrients. In many cases nitrogen is the limiting essential element. Nitrogen shortage causes detrimental effects on agricultural productivity, yet excessive nitrogen fertilization accounts for negative economic and environmental impacts. Improving nitrogen use efficiency represents a main challenge for agriculture, and it becomes increasingly important to investigate the mechanisms of nitrogen uptake, storage and recycling and to understand the interplay of these processes with the regulation of plant development and stress defense.

Due to the highest nitrogen to carbon ratio among the 21 proteinogenic amino acids, arginine is a major storage and transport form for organic nitrogen in plants in addition to its role as an amino acid for protein synthesis, a precursor for polyamines and nitric oxide (NO) and an essential metabolite for many cellular and developmental processes. In seed proteins of different plant species 40–50% of the total nitrogen reserve is represented by arginine (VanEtten et al., 1963; King and Gifford, 1997), and this amino acid accounts for 50% of the nitrogen in the free amino acid pool in developing embryos of soybean (Micallef and Shelp, 1989) and pea (de Ruiter and Kollöffel, 1983). Arginine is often a major nitrogen storage form also in underground storage organs and roots of trees and other plants (Nordin and Näsholm, 1997; Bausenwein et al., 2001; Rennenberg et al., 2010). Therefore, arginine metabolism plays a key role in nitrogen distribution and recycling in plants (Slocum, 2005).

Slocum (2005) reviewed those genes that have been identified as encoding enzymes involved in arginine synthesis in Arabidopsis (*Arabidopsis thaliana*) and presented the current state of their characterization, including subcellular targeting, gene expression, available mutants and cDNAs of each enzyme. Over the past 10 years, research mainly on Arabidopsis as model plant has generated significant progress in our understanding of arginine metabolism, whereas several crucial questions remain unanswered. The present review highlights challenges for future research on plant arginine metabolism by summarizing recent advances about biosynthesis, distribution and catabolism of arginine and its contribution to polyamine and NO synthesis.

## Arginine Biosynthesis

The biosynthetic pathway of arginine can be divided in two processes. First, ornithine is synthesized from glutamate either in a cyclic or a linear pathway, followed by the synthesis of arginine from ornithine.

## Cyclic and Linear Pathways for Ornithine Synthesis

**FIGURE 1:**
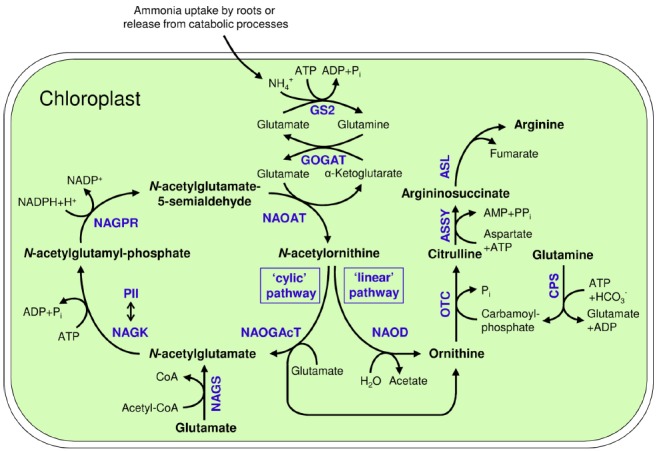
**Arginine biosynthesis in plastids and its connection to ammonium assimilation by the GS2/GOGAT-system; GS2: Glutamine synthetase 2; GOGAT: Glutamate synthase; NAGS: ***N***-acetylglutamate synthase; NAGK: ***N***-acetylglutamate kinase; NAGPR: ***N***-acetylglutamatyl-5-P reductase; NAOAT: ***N***-acetylornithine aminotransferase; NAOGAcT: ***N***-acetylornithine-glutamate acetyltransferase; NAOD: ***N***-acetylornithine deacetylase; OTC: Ornithine transcarbamylase; ASSY: Argininosuccinate synthase; ASL: Argininosuccinate lyase; CoA: Coenzyme A; CPS: Carbamoyl phosphate synthetase; NAGK/PII double headed arrow: Regulatory interaction of NAGK and PII protein**.

Ornithine is synthesized from glutamate *via* several acetylated intermediates (Figure 1). In the first step, *N*-acetylglutamate synthase (NAGS) uses acetyl-coenzyme A (Acetyl-CoA) to transfer an acetyl moiety to glutamate forming *N*-acetylglutamate (Slocum, 2005). *N*-acetylglutamate is then phosphorylated at the C5 position by *N*-acetylglutamate kinase (NAGK). The next step, the formation of *N*-acetylglutamate-5-semialdehyde (NAcGSA), is catalyzed by *N*-acetylglutamate-5-P reductase (NAGPR). In the fourth step, an amino group is transferred from a second glutamate molecule to *N*-acetylglutamate-5-semialdehyde by *N*^2^-acetylornithine aminotransferase (NAOAT), yielding *N*^2^-acetylornithine. Subsequently, ornithine is released by transferring the acetyl residue to glutamate by *N*-acetylornithine:*N*-acetylglutamate acetyltransferase (NAOGAcT), giving this enzyme the key role of conserving the acetyl group for the next cycle of ornithine synthesis. NAOGAcT is found in non-enteric bacteria (Cunin et al., 1986), fungi (Davis, 1986), and plants (Shargool et al., 1988).

*Escherichia coli* and other enterobacteria, as well as yeast, synthesize ornithine in a linear pathway due to the presence of *N*-acetylornithine deacetylase (NAOD), which hydrolyses *N*-acetylornithine to ornithine and acetate as the final step (Vogel and Bonner, 1956; Meinnel et al., 1992; Crabeel et al., 1997). Plants were considered as unable to use this pathway, since NAOD activity has not been demonstrated in plants so far (Slocum, 2005; Page et al., 2012; Frémont et al., 2013). Recently, Molesini et al. (2015) revealed the first hint of NAOD activity in Arabidopsis using T-DNA insertion lines (see below).

Glutamate is a precursor for both proline and ornithine biosynthesis in plants and other organisms (Morris et al., 1969; Cunin et al., 1986; Davis, 1986; Caldovic and Tuchman, 2003). After acetylation of glutamate, the NAcGSA intermediate is incapable of undergoing cyclisation. Thus, acetylation of glutamate commits it to ornithine synthesis. In contrast, in proline biosynthesis the corresponding non-acetylated glutamate-5-semialdehyde (GSA) spontaneously forms pyrroline-5-carboxylate (P5C) by cyclisation (Slocum, 2005).

### Arginine Synthesis from Ornithine

Arginine is synthesized from ornithine by the enzymes of the linear “arginine pathway” (Micallef and Shelp, 1989; Slocum, 2005). Ornithine transcarbamoylase (OTC) delivers the third N-atom by carbamoylation of the δ-amino group of ornithine, forming citrulline. This reaction requires carbamoyl phosphate, which is generated from ATP, bicarbonate and the δ-amino group of glutamine by carbamoyl phosphate synthetase (CPS). The fourth N-atom of arginine is derived from aspartate, which is ligated to citrulline by argininosuccinate synthase (ASSY). As substrates of OTC and ASSY, the amino acids aspartate and glutamine are additional essential precursors for arginine synthesis. Finally, argininosuccinate lyase (ASL) splits off fumarate, generating the final product arginine (Slocum, 2005; Figure 1).

The enzymes of plant arginine biosynthesis have been partly characterized biochemically (Shargool et al., 1988), but still little is known about the genes encoding these enzymes, and many steps of arginine biosynthesis remain poorly characterized in plants.

### Genes and Enzymes of Arginine Biosynthesis and their Regulation

#### NAGS

The first, and presently the only, characterized plant NAGS was isolated from tomato (Kalamaki et al., 2009). *SlNAGS1* is a single copy gene and the *Sl*NAGS protein shows a high level of similarity to two predicted Arabidopsis NAGS proteins, NAGS1 (At2g22910) and NAGS2 (At4g37670). A plastid transit peptide is predicted for *Sl*NAGS1 and the plastid localization is supported by the expression of *SlNAGS1* in all aerial organs, whereas no expression was detected in roots (Kalamaki et al., 2009). Transgenic Arabidopsis plants overexpressing *SlNAGS1* showed a significant accumulation of ornithine in the leaves, and a higher tolerance to salt and drought stress compared to wild type plants. The improved tolerance to salt stress of *SlNAGS1* overexpressions was attributed to the elevated levels of ornithine, citrulline and arginine, since these amino acids have been reported to accumulate together with proline in higher plants under salinity stress (Mansour, 2000; Ashraf and Harris, 2004).

NAGS activity is a target of feedback regulation by arginine in prokaryotes and a similar mechanism is proposed for plant NAGS (Kalamaki et al., 2009; Sancho-Vaello et al., 2009). Sancho-Vaello et al. (2009) showed regulation of *Pseudomonas aeruginosa* NAGS activity by arginine, being an activator at low arginine concentration as well as an inhibitor at higher arginine concentration. The effects of arginine on NAGS activity were mediated by altering domain interactions within NAGS.

#### NAGK

The localization of Arabidopsis NAGK (At3g57560) in chloroplasts was predicted by sequence analysis and was experimentally demonstrated by Chen et al. (2006). Feedback regulation of NAGK mediated by the plastidic PII protein was first described in the cyanobacterium *Synechococcus elongatus* (Heinrich et al., 2004; Maheswaran et al., 2004) and in Arabidopsis (Burillo et al., 2004). PII proteins are among the most highly conserved, widely distributed and ancient signal transduction proteins known in bacteria, archaebacteria, cyanobacteria, eukaryotic algae and higher plants. They are involved in sensing the carbon/nitrogen balance and the energy status of the cell. Targets include signal transduction proteins, key metabolic enzymes and transporters involved in nitrogen assimilation and uptake (Slocum, 2005; Feria Bourrellier et al., 2009). The PII protein has been shown to interact tightly with NAGK, inducing a conformational change of its T-loop and leading to decreased feedback inhibition of the enzyme complex by arginine (Heinrich et al., 2004; Slocum, 2005; Llacer et al., 2008; Feria Bourrellier et al., 2009). Interaction of NAGK and the PII protein under conditions of high nitrogen availability strongly increases the catalytic efficiency of NAGK and decreases significantly the sensitivity of the enzyme complex to arginine, resulting in high arginine production. Limitation of nitrogen prevents the formation of the NAGK/PII complex, resulting in decreased enzyme activity of NAGK and increasing the feedback inhibition of the complex by arginine (Heinrich et al., 2004; Maheswaran et al., 2004; Slocum, 2005; Chen et al., 2006; Llacer et al., 2008). Feria Bourrellier et al. (2009) demonstrated that the interaction of NAGK and the PII protein was counteracted by α-ketoglutarate/2-oxoglutarate, the carbon skeleton used to form glutamate during nitrogen assimilation and a low-nitrogen abundance signal in plants (Lancien et al., 2000), as well as by arginine and glutamate. The flux through the arginine biosynthetic pathway depends on the balance between energy status and nitrogen and carbon availability for nitrogen assimilation *via* the glutamine synthetase (GS)/glutamate synthase (GOGAT) pathway in the plastids. Schneidereit et al. (2006) showed a threefold decrease in the intracellular α-ketoglutarate level induced by high nitrogen conditions in plants, due to a rapid NH_4_^+^ assimilation by the GS/GOGAT cycle. This suggests that a high nitrogen status will be sensed by the PII protein through a low level of α-ketoglutarate, and thus under these conditions PII-NAGK complex formation will be favored leading to arginine synthesis and nitrogen storage, as well as an increase in arginine and glutamate concentrations, which are expected to limit arginine accumulation by inhibition of NAGK (Feria Bourrellier et al., 2009). Chellamuthu et al. (2014) identified an additional PII-mediated regulatory mechanism, by which high nitrogen availability activates NAGK and thus promotes arginine synthesis. Glutamine binding alters PII conformation, promoting the interaction with and activation of NAGK. This mechanism appears to be conserved from algae to flowering plants with the exception of the Brassicaceae, including Arabidopsis.

#### NAGPR, NAOAT, and NAOGAcT

Since Slocum (2005) NAGPR has not been characterized further in Arabidopsis. Rice NAGPR was crystallized and characterized (Nonaka et al., 2005). The crystal structure of a putative NAGPR from Arabidopsis (At2g19940) was deposited in the protein data bank (www.rcsb.org/pdb; PDB accession number #1XYG; Levin et al., 2007). However, the details of these structures have not been reported so far.

The NAOAT encoding gene *Arg9* was identified and characterized in the green alga *Chlamydomonas reinhardtii* (Remacle et al., 2009). Plastidial localization of NAOAT was demonstrated by complementation studies as well as immunoblot analysis. The *TUMOR PRONE5* (*TUP5*, At1g80600) gene of Arabidopsis was demonstrated to encode a NAOAT (Frémont et al., 2013). Characterization of the gene and its mutant lines showed a strongly reduced free arginine content in the chemically-induced recessive mutant *tup5*, suggesting that the biosynthesis of amino acids that are produced downstream of the NAOAT enzymatic reaction is impaired in this mutant. Consistently, *tup5* showed a short root growth phenotype, restorable by supplementation with arginine and its metabolic precursors. A yeast NAOAT mutant was complemented by *TUP5*. Two null alleles of *TUP5* showed a reduced viability of gametes and embryo lethality, possibly caused by insufficient arginine supply from maternal tissue. A TUP5-green fluorescent protein was localized in chloroplasts (Frémont et al., 2013). *TUP5* expression is positively regulated by light, and *tup5* showed a unique light-dependent short root phenotype. The roots of *tup5* seedlings of different ages cultivated in darkness immediately stopped growth when they were shifted into light. Frémont et al. (2013) attributed this phenotype to a blue light-dependent switch from indeterminate growth to determinate growth with arresting cell production and an exhausted root apical meristem and, thus, a critically dependence of root growth on arginine in the presence of light.

No experimental analysis of the putative Arabidopsis NAOGAcT (At2g37500) has been described yet. Expression of the poplar NAOGAcT homolog was not altered in response to putrescine overproduction in a transgenic line (Page et al., 2012).

#### NAOD

NAOD activity has never been demonstrated in plants, although many putative NAOD-like genes have been identified (Slocum, 2005). Molesini et al. (2015) analyzed the NAOD-activity in Arabidopsis after downregulation of the putative NAOD gene (At4g17830) by using RNA silencing and T-DNA insertion mutants. All analyzed NAOD-suppressed plants showed consistently reduced ornithine content compared with wild-type plants, suggesting that in addition to NAOGAcT action, NAOD contributes to the regulation of ornithine levels in plant cells. Ornithine depletion was associated with increased putrescine and decreased spermine concentrations, and the reduced *AtNAOD* expression resulted in developmental alterations, namely early flowering and impaired seed setting. A connection between ornithine levels or metabolism and reproductive development had already been proposed by Trovato et al. (2001), who observed early flowering and enhanced flower formation in tobacco plants overexpressing ornithine cyclodeaminase (RolD) from *Agrobacterium rhizogenes* (see below).

#### OTC, CPS, ASSY, and ASL

Quesada et al. (1999) described a transfer DNA (T-DNA) insertion mutant of Arabidopsis with an insertion downstream of the OTC (At1g75330) open reading frame. The mutant plants showed an increased sensitivity to exogenous ornithine, which was attributed to reduced OTC expression, potentially due to problems in mRNA 3′-end formation (Quesada et al., 1999). The chemically-induced Arabidopsis mutants *ven3* and *ven6*, where the small subunit (At3g27740) and the large subunit (At1g29900) of CPS were affected, showed increased ornithine and decreased citrulline levels, respectively, suggesting a disrupted conversion of ornithine to citrulline because of reduced carbamoyl phosphate availability (Mollá-Morales et al., 2011). We could not find any further recent publication reporting on the characterization of plant OTC or ASSY (At4g24830).

A characterization of the Arabidopsis ASL (At5g10920) is also still missing. The rice ASL mutant *osred1* showed a short root phenotype like the Arabidopsis NAOAT mutant *tup5-1*, supporting the suggestion that arginine is essential for normal root growth in different plant species (Frémont et al., 2013; Xia et al., 2014a,b). Expression analysis revealed two alternatively spliced transcripts of *OsASL1*, *OsASL1.1*, and *OsASL1.2*, coding for two ASL isoforms with slightly different N-termini. *OsASL1.1* was expressed throughout the entire growth period in most organs, whereas *OsASL1.2* was expressed mainly in the roots. In contrast to the plastid-localized OsASL1.1, OsASL1.2 was localized in the cytosol and nucleus. Only OsASL1.1 showed ASL activity in a yeast complementation study. The short-root phenotype of the *osred1* mutant was rescued by external arginine supply but not by a NO donor, supporting the hypothesis that arginine is required for normal root growth independently of its function as putative NO precursor (Xia et al., 2014a,b). The poplar *ASL* homolog was the only gene, among 17 analyzed genes of arginine metabolism in poplar, whose expression was higher in response to putrescine overproduction in a transgenic line. Page et al. (2012) hypothesized a biochemical regulation of arginine biosynthesis involving substrate concentrations or co-factors rather than a regulation at the transcriptional level.

## Arginine Transport

### Long Distance Transport

Long distance transport of arginine to nitrogen storing organs or seeds occurs probably in the vascular tissue and is presumably dependent on amino acid transporters of the AAP family of amino acid/proton co-transporters. Especially important for long distant arginine transport seem to be AAP3 (At1g77380) and AAP5 (At1g44100), which are involved in loading and unloading the vascular tissue (Fischer et al., 1995, 2002; Okumoto et al., 2004; Svennerstam et al., 2008; Tegeder, 2014). AAP5 transports arginine and lysine with high affinity (Svennerstam et al., 2008) and seems to have an important role in the uptake of basic amino acids by roots (Svennerstam et al., 2011). An additional function of AAP5 in the transport of arginine within plants is supported by its expression throughout the entire vascular system of Arabidopsis (Fischer et al., 1995, 2002; Svennerstam et al., 2008). AAP3 also displays high affinity for basic amino acids (Fischer et al., 2002; Taylor et al., 2015) and was shown to be expressed in the phloem, predominantly in roots (Okumoto et al., 2004). AAP transporters have been localized to the collection phloem of legumes and they are predicted to play a major role in amino acid loading of this tissue. In Arabidopsis, the assignment of clear-cut physiological function to individual AAPs has not been reported so far (Tegeder, 2014).

Dündar and Bush (2009) identified and characterized a bidirectional amino acid transporter (BAT1, At2g01170) in Arabidopsis. Both direct measurement of amino acid transport and yeast growth experiments demonstrated transport activity of BAT1 for alanine, arginine, glutamate and lysine. *BAT1* is a single copy gene in the Arabidopsis genome and its mRNA is ubiquitously produced in all organs. Promoter-GUS analysis localized *BAT1* expression in the vascular tissue, suggesting that BAT1 may function in amino acid export from the phloem into sink tissues (Dündar and Bush, 2009).

### Intracellular Transport

Arginine metabolism is distributed over the three cellular compartments cytosol, plastids and mitochondria. Newly synthesized arginine can be used for protein synthesis directly in plastids or, after intracellular transport, in the cytosol and mitochondria. This generates a need for transport systems for arginine, as well as for synthesis and degradation intermediates. Very little is known about the transport of amino acids into or out of chloroplasts. Members of the preprotein and amino acid transporter (PRAT) family were proposed to mediate transport of amino acids across the inner envelope membrane (Murcha et al., 2007; Pudelski et al., 2010). So far, experimental evidence is only available for the function of PRATs in protein import (Rossig et al., 2013).

The prevalent group of carrier proteins in mitochondria is the mitochondrial carrier family (MCF) with 58 putative members in Arabidopsis (Picault et al., 2004; Haferkamp and Schmitz-Esser, 2012). Two members of the MCF were identified as basic amino acid transporters (BAC1 and BAC2) which mediate the transport of arginine, ornithine and lysine with decreasing affinity and were postulated to be localized in the mitochondrial inner membrane (Hoyos et al., 2003; Palmieri et al., 2006). BAC1 (At2g33820) and BAC2 (At1g79900), together with BOU (*a bout de souffle*, Lawand et al., 2002), form a sub-group of MCF proteins distinct from other Arabidopsis mitochondrial carriers regarding sequences and function (Catoni et al., 2003; Hoyos et al., 2003; Picault et al., 2004; Toka et al., 2010).

BAC1 and BAC2 were identified as basic amino acid transporters by complementation of the yeast mutant *arg11*. This mutant is defective in mitochondrial ornithine/arginine transport due to a loss-of-function mutation in the ORT1 carrier (Catoni et al., 2003; Hoyos et al., 2003; Palmieri et al., 2006). ORT1 is an antiporter for ornithine, arginine or lysine and is important for ornithine export from mitochondria, an essential step for arginine biosynthesis in Saccharomyces cerevisiae (Palmieri et al., 1997, 2006). The transport characteristics of BAC1 and BAC2 resemble each other, they were inactivated by the same inhibitors and their K_m_ and V_max_ values were very similar for their most efficiently transported and preferred substrate arginine (Palmieri et al., 2006).

Arabidopsis *bac2* mutants showed a conditional phenotype as they grew more slowly than the wild-type on arginine as sole source of nitrogen, while *BAC2* overexpressing plants showed the opposite phenotype. Presumably, the expression of BAC2 is a limiting factor for mitochondrial arginine transport *in vivo* and therefore for the mobilization of nitrogen from arginine (Toka et al., 2010). This is consistent with the higher expression levels of BAC2 in wild type seedlings growing on arginine as sole source of nitrogen (Catoni et al., 2003).

Since *bac2* mutants did not show any phenotypical difference to the wild type when growing on soil, other pathways or transporters seem to compensate the lack of BAC2 during vegetative growth (Toka et al., 2010). BAC2 expression was induced during stress and senescence (Toka et al., 2010) and *bac2* mutant seedlings recovering from hyperosmotic stress showed significantly reduced leaf growth (Planchais et al., 2014). Probably BAC2-dependent arginine import into mitochondria is required during stress conditions and for recovery of growth after stress (Planchais et al., 2014). The functional redundancy between BAC1 and BAC2 and the expression patterns indicate that BAC1 is sufficient for mitochondrial arginine import during normal plant growth (Catoni et al., 2003; Hoyos et al., 2003; Toka et al., 2010). So far, no information about *bac1* knock-out mutants or phenotypes of BAC1 overexpressing plants is available, leaving this point speculative.

Like in many other plant species, storage of nitrogen in the form of arginine in seeds is also likely in Arabidopsis, since the total arginase activity, which initiates the release of nitrogen from arginine, increased strongly up to 6 days after germination accompanied by increases in free arginine and urea levels (Zonia et al., 1995). In order to degrade arginine stored in seeds for nitrogen remobilization, large amounts of arginine have to be transported into mitochondria by the BAC carriers during early seedling development making it accessible to the mitochondrial localized arginase (Flores et al., 2008). However, only low levels or no expression of BAC2 were found in seeds and seedlings of Arabidopsis (Hoyos et al., 2003; Toka et al., 2010). This finding argues against a prominent function of BAC2 in storage mobilization, indicating that another transporter, probably BAC1, mediates the import of arginine into mitochondria during early seedling development. This suggestion is supported by RT-PCR analysis of BAC1, which is highly expressed in seedlings, contrary to BAC2, which is mostly expressed in stamens and pollen grains of flowers (Hoyos et al., 2003; Palmieri et al., 2006; Toka et al., 2010; Monné et al., 2015).

Amino acid analysis revealed accumulation of proline and alanine in *bac2* mutants (Planchais et al., 2014). BAC2 overexpressing plants showed low arginine levels and simultaneously high levels of ornithine, urea and citrulline, all products of arginine catabolism. Thus, BAC2 is able to increase arginine availability for degradation inside mitochondria, especially under stress conditions (Toka et al., 2010; Planchais et al., 2014; Monné et al., 2015).

## Arginine Catabolism and Arginine-Derived Metabolites

### Arginine Catabolism

**FIGURE 2:**
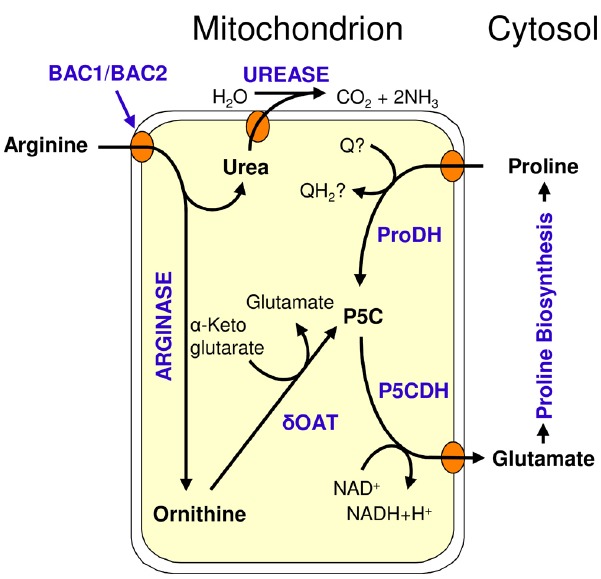
**Catabolism of arginine in Arabidopsis; BAC1/BAC2: Basic amino acid transporter 1/2; δOAT: Ornithine-δ-aminotransferase; ProDH: Proline dehydrogenase; P5CDH: P5C dehydrogenase; P5C: Pyrroline-5-carboxylate; Q/QH_2_: oxidized/reduced ubiquinone**.

After the import of arginine by BAC1 and BAC2 into mitochondria, arginine catabolism starts with degradation of arginine to ornithine and urea by arginase (Figure 2). Urea is exported to the cytosol, where it is further degraded to ammonia by urease (Witte, 2011; Polacco et al., 2013). Ornithine could be transported back into plastids to re-enter arginine biosynthesis as in the mammalian urea cycle. However, cycling between ornithine and arginine is unlikely to occur in a single cell or tissue in plants, as it would constitute a waste of energy and assimilated nitrogen. Ornithine degradation proceeds by transfer of the δ-amino group to α-ketoglutarate, catalyzed by ornithine δ-aminotransferase (δOAT), yielding GSA/P5C, and glutamate. GSA/P5C is subsequently converted to a second molecule of glutamate by P5C dehydrogenase (P5CDH). Glutamate is either exported from mitochondria as an anabolic precursor for multiple pathways, or it is further degraded inside mitochondria to α-ketoglutarate, ammonium and NADH by glutamate dehydrogenase (GDH). NADH can be used to fuel respiratory ATP production, while α-ketoglutarate can be fed into the citric acid cycle or can be used to re-assimilate ammonium in the GS/GOGAT system.

Like arginine synthesis, arginine catabolism is also regulated in accordance with the overall nutritional status of the plant cell. Arginine utilization seems to be coordinated with the availability of carbohydrates, since sugar starvation caused a substantial increase of enzyme activities of arginase and urease, as well as arginine decarboxylase (ADC), starting polyamine synthesis (see below) in yellow lupin (*Lupinus luteus* L.; Borek et al., 2001).

In the following sections, we will firstly summarize the available data on the single steps in the degradation of arginine to glutamate and secondly we will discuss alternative metabolic routes using arginine as a precursor.

#### Arginase

Arginase (L-arginine ureahydrolase or amidohydrolase) catalyzes the initial step of arginine degradation and is a binuclear manganese metalloenzyme (Goldraij and Polacco, 2000; Todd et al., 2001). Crystal structures of arginases revealed a homotrimeric quaternary structure for rat arginase 1 (Kanyo et al., 1996), human arginase 1 (Di Costanzo et al., 2007) and human arginase 2 (Cama et al., 2003), as well as homo-hexameric structures of *Bacillus caldovelox* (Bewley et al., 1999) and *Thermus thermophilus* (Dowling et al., 2008) arginases. Arginases have been purified and characterized from different plant species such as soybean (Kang and Cho, 1990), iris bulbs (Boutin, 1982), peanut seedlings (Desai, 1983), oakmoss (Martín-Falquina and Legaz, 1984) and ginseng (Hwang et al., 2001), showing variable sizes of monomers and oligomers. For arginase purified from Catjang (*Vigna unguiculata* var. *cylindrica*), a native molecular weight of 210 kDa was estimated, while the monomer had an apparent molecular weight of 52 kDa (Dabir et al., 2005). This supports a tetrameric structure of arginase from Catjang. In contrast, arginase purified from ginseng was estimated to be decameric (Hwang et al., 2001).

Arabidopsis contains two arginase genes, *ARGAH1* (At4g08900) and *ARGAH2* (At4g08870), which probably arose by a recent gene duplication (Krumpelman et al., 1995; Brownfield et al., 2008). The predicted mature proteins show 91% sequence identity, whereas the predicted mitochondrial transit peptide share only 39% sequence identity. GFP-fusion proteins showed that both ARGAH1 and ARGAH2 are mitochondrial proteins (Palmieri et al., 2006; Flores et al., 2008). The formation of homo- and hetero-oligomers of the two Arabidopsis arginase isoforms has been demonstrated, while the precise oligomeric state and three-dimensional structure remain to be resolved (Winter, 2013).

Seedling arginase activity increases sharply during germination in Arabidopsis (Zonia et al., 1995), loblolly pine (King and Gifford, 1997) and other plant species (Splittstoesser, 1969; Kollöffel and van Dijke, 1975; Kang and Cho, 1990; Goldraij and Polacco, 1999). The analysis of T-DNA insertion mutants demonstrated that roughly 85% of the arginase activity in Arabidopsis seedlings depends on ARGAH2 whereas no developmental defects of *argah2* or *argah1* mutants were reported (Flores et al., 2008). Infection of mature Arabidopsis plants with the necrotrophic fungus *Botrytis cinerea* or the protist *Plasmodiophora brassicae*, causing the agriculturally important clubroot disease, resulted in an upregulation of ARGAH2 expression. Consistently, *argah2* mutants showed an increased sensitivity toward clubroot disease (Brauc et al., 2012; Gravot et al., 2012).

Conversely to increased pathogen susceptibility, *argah1-1* and *argah2-1* T-DNA insertion mutants as well as the double mutant *argah1argah2* showed increased tolerance to abiotic stress. Higher tolerance to water deficit, salt stress and freezing was accompanied by increased NO and polyamine accumulation (Flores et al., 2008; Shi et al., 2013). Consistently, overexpression of arginase in Arabidopsis decreased the resistance and defense against abiotic stress (Shi et al., 2013). No developmental defects were reported for the *argah1argah2* double mutants under normal growth conditions.

Interestingly, mutation of the single copy arginase gene in rice caused a strong decrease in growth and fertility, affecting both grain size and the rate of seed setting, whereas overexpression of arginase improved yield under nitrogen-limiting conditions (Ma et al., 2012).

### Urea and Urease

Arginase activity is the main source for endogenous urea in higher plants, and recycling of urea seems to be especially important under stress conditions. The available information about metabolism and transport of urea in plants has recently been reviewed by Witte (2011) and Polacco et al. (2013). Urease (urea amidohydrolase) is the only known Ni-containing enzyme in plants (Dixon et al., 1975) and catalyzes the hydrolysis of urea to ammonia and carbamic acid; the latter spontaneously hydrolyzes to ammonia and bicarbonate (Figure 2). The functional assembly of Arabidopsis urease (At1g67550) requires at least three accessory proteins (Witte et al., 2005; Witte, 2011). In Arabidopsis, the production of urea is induced by jasmonic acid by upregulation of ARGAH2 expression (Brownfield et al., 2008). The rapid hydrolysis of urea by urease could cause localized alkalinisation, which, in turn, could further stimulate arginase which has a pH optimum ≥9.5 (Jenkinson et al., 1996; Polacco et al., 2013). The alkalinization of the cytosol by induction of arginase and urease activity might constitute an active component of pathogen defense mechanisms (Polacco et al., 2013).

There is a large flux of nitrogen from arginine to ammonia pools due to arginase and urease activity, especially during germination. The increases in free urea levels during germination are generally rather moderate, indicating that urea export from mitochondria and urease are not limiting for nitrogen re-mobilization (Polacco et al., 2013). In addition to germination, urease plays a key role in recycling of nitrogen stored as arginine during senescence or during seasonal changes.

#### Ornithine δ-Aminotransferase

The second product of arginine hydrolysis is ornithine, which is catabolized by δOAT to GSA and glutamate (Figure 2). δOAT transfers the δ-amino group of ornithine to α-ketoglutarate and the equilibrium of the δOAT reaction has been found far on the GSA + glutamate side (Adams and Frank, 1980).

A direct contribution of δOAT to stress-induced proline accumulation, which would require an unknown exit route of mitochondrial GSA or P5C to the cytosol, where P5C reductase (P5CR) is localized, is controversial (Stránská et al., 2008). Proline production by the reverse reaction of proline dehydrogenase (ProDH) is energetically unfavorable. Furthermore, due to the chemical instability of GSA/P5C (Williams and Frank, 1975) and its toxicity when accumulating (Deuschle et al., 2004), export from mitochondria to the cytosol and thus contribution to proline synthesis seems unlikely, but cannot be fully excluded. Roosens et al. (1998) hypothesized that Arabidopsis δOAT (At5g46180) plays an important role in proline accumulation during osmotic stress in plants, because of increased free proline content, δOAT activity and *δ*OAT mRNA in young plantlets under salt stress conditions. This hypothesis was supported by the analysis of transgenic *Nicotiana plumbaginifolia* plants overexpressing Arabidopsis δOAT, which synthesized more proline than the control plants and showed a higher biomass and a higher germination rate under osmotic stress conditions (Roosens et al., 2002). The exclusive targeting of δOAT to mitochondria in Arabidopsis and unchanged proline accumulation in salt-stressed δ*oat* knockout mutants provided strong evidence against a direct contribution of δOAT to stress-induced proline accumulation (Funck et al., 2008). However, δOAT activity was correlated to proline accumulation in salt-stressed cashew plants and ornithine application strongly enhanced proline accumulation (da Rocha et al., 2012). Overexpression of δOAT in rice resulted in higher proline levels and activated the antioxidant defense, rendering the plants more stress-tolerant (You et al., 2012).

Silencing or deletion of *δOAT* in tobacco and Arabidopsis, respectively, compromised non-host pathogen defense and pathogen-induced ROS formation (Senthil-Kumar and Mysore, 2012). Further research is needed to understand how δOAT contributes to stress defense and whether GSA produced by δOAT can be used directly for proline synthesis or is obligatorily converted to glutamate by P5CDH.

#### P5C Dehydrogenase

GSA produced by δOAT inside mitochondria is most probably further converted to glutamate by mitochondrial P5CDH (Deuschle et al., 2001; Funck et al., 2008). Due to the mentioned chemical instability and toxicity of GSA/P5C, the formation of a reversible enzyme complex of δOAT and P5CDH seems likely, channeling GSA to P5CDH without releasing it to the mitochondrial matrix (Elthon and Stewart, 1982; Funck et al., 2008). Substrate channeling from ProDH to P5CDH has been reported for the bifunctional enzyme PutA from *Geobacter sulfurreducens* and recently also for the monofunctional enzymes from *Thermus thermophilus* (Singh et al., 2014; Sanyal et al., 2015). A similar co-operation between ProDH, δOAT and P5CDH in plants might explain the relatively low affinity of isolated P5CDH for P5C (around 0.5 mM) as opposed to the fivefold higher affinity of P5C reductase (Forlani et al., 1997a,b; Giberti et al., 2014). Consistently, two P5CDH isoforms have been detected in *Nicotiana plumbaginifolia*, that may be specifically involved in the oxidation of P5C deriving from either proline or arginine (Forlani et al., 1997a). The Arabidopsis genome contains a single *P5CDH* gene (At5g62530) and knockout mutants were hypersensitive to external supply of arginine or ornithine (Deuschle et al., 2004). In tissues with a high energy demand, glutamate produced by δOAT and P5CDH may be further degraded by GDH to fuel mitochondrial energy production. However, since GDH also releases ammonia, which needs to be re-assimilated in an energy-demanding process, a direct recycling of glutamate for anabolic pathways seems more likely.

### Arginine as Precursor for Proline Biosynthesis

Feeding of plants with arginine or ornithine resulted in elevated proline levels and radiotracer experiments demonstrated that both ^3^H and ^14^C from arginine can be recovered as proline (Adams and Frank, 1980; da Rocha et al., 2012). The physiological relevance and the biochemical pathway of the conversion of arginine to proline in plants remain unclear. The most prominent hypothesis is that ornithine, derived from arginine catabolism, is converted by δOAT to GSA/P5C, which then serves as substrate for proline synthesis by P5CR. This model has been doubted, since Arabidopsis δOAT was found to be exclusively localized in mitochondria, while P5CR is localized in the cytosol (Funck et al., 2008, 2012). Isolated corn mitochondria incubated with proline or ornithine released very little P5C and this release was strongly pH dependent and stimulated upon swelling of the mitochondria (Elthon and Stewart, 1982). Direct export of P5C from mitochondria and conversion to proline by P5C reductase were postulated as part of a reactive oxygen species-producing proline-P5C cycle (Miller et al., 2009). Direct evidence for the transport of P5C and the operation of this cycle under physiological conditions is still missing. Inside mitochondria, GSA/P5C is further converted to glutamate by P5CDH (see above). Export of glutamate from mitochondria has been demonstrated and could be the basis for proline synthesis *via* the glutamate pathway (Linka and Weber, 2005; Di Martino et al., 2006).

Removal of the α-amino group of ornithine and conversion of the resulting pyrroline-2-carboxylate to proline has also been proposed (Mestichelli et al., 1979), but the required enzymes were not described in plants to date. Another alternative pathway from ornithine to proline would be *via* ornithine cyclodeaminase (OCD), which is found in bacteria and is transferred into plants with the T-DNA of *Agrobacterium rhizogenes* (Trovato et al., 2001; Mattioli et al., 2008). In the Arabidopsis genome, a homolog (At5g52810) of bacterial OCDs and mammalian μ-crystallins has been identified. However, plants with a decreased expression of the putative OCD had higher rather than lower proline levels and the analysis of the recombinant protein yielded no evidence for OCD activity (Sharma et al., 2013).

### Polyamine Synthesis from Arginine

Polyamines (putrescine, spermidine, and spermine) are essential for development and stress responses of plants. Embryogenesis, organogenesis, particularly flower initiation and development, fruit setting and ripening, as well as leaf senescence all require polyamines (Page et al., 2012; Majumdar et al., 2013; Pathak et al., 2014). In addition, the role of polyamines for abiotic stress tolerance and the regulation of nitrogen assimilation is well established in plants. Accumulation of polyamines in large amounts in the cell points toward roles in metabolic regulation of ammonia toxicity, NO production, and balancing organic nitrogen metabolism in the cell (Maiale et al., 2004; Marco et al., 2011; Gupta et al., 2013; Guo et al., 2014; Minocha et al., 2014; Pathak et al., 2014; Tiburcio et al., 2014).

**FIGURE 3:**
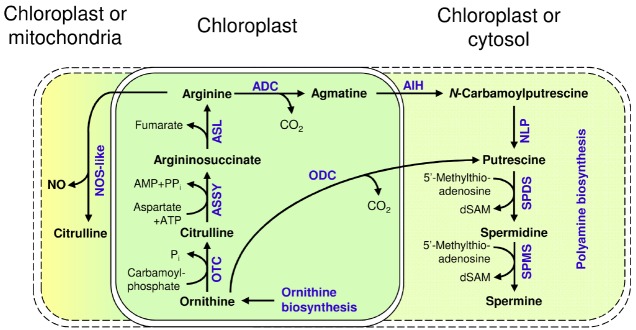
**Metabolism of arginine and its utilization for polyamine synthesis and NO generation in Arabidopsis; ODC: Ornithine decarboxylase; OTC: Ornithine transcarbamylase; ASSY: Argininosuccinate synthase; ASL: Argininosuccinate lyase; ADC: Arginine decarboxylase; AIH: Agmatine iminohydrolase; NLP: ***N***-carbamoylputrescine amidase; SPDS: Spermidine synthase; SPMS: Spermine synthase; dSAM: decarboxylated ***S***-Adenosyl-L-methionine; NOS-like: Nitric oxide synthase like; NO: Nitric oxide**.

Arginine is a precursor for the synthesis of polyamines (Bagni and Tassoni, 2001; Illingworth et al., 2003) and polyamine biosynthesis in Arabidopsis begins with arginine, which is converted by arginine decarboxylase (ADC1: At2g16500 and ADC2: At4g34710) to agmatine. ADC was found to be localized in the chloroplast of oat (*Avena sativa*, Borrell et al., 1995), which is consistent with the biosynthesis of arginine in the chloroplasts. The enzymatic reaction of ADC is followed by agmatine iminohydrolase (AIH, At5g08170, Janowitz et al., 2003) and *N*-carbamoylputrescine amidase (NLP, At2g27450, Piotrowski et al., 2003) producing putrescine. Subsequently spermidine and spermine are formed by two different aminopropyltransferases, spermidine synthase (SPDS, At1g23820, At1g70310) and spermine synthase (SPMS, At1g23820, At1g70310, At5g53120; reviewed in, Shao et al., 2012) with so far unknown localization (Figure 3).

Ornithine decarboxylase (ODC) represents an alternative way for putrescine synthesis by decarboxylation of ornithine. ODC homologs were identified and analyzed in different plant species including datura (Michael et al., 1996), tomato (Alabadí and Carbonell, 1998), tobacco (Lee and Cho, 2001), and chilli (Zainal et al., 2002). The Arabidopsis genome probably lacks a recognizable ODC (Hanfrey et al., 2001). This is in apparent contradiction to the findings of Molesini et al. (2015), who demonstrated that a lowered ornithine level in Arabidopsis NAOD insertion mutants did not influence arginine content but affected the levels of polyamines. In turn, this is consistent with the findings of Majumdar et al. (2013), who analyzed the regulation of ornithine and ornithine-related pathways (arginine and polyamines) in Arabidopsis by diversion of ornithine from arginine biosynthesis, *via* the overexpression of a mouse ODC. The removal of large amounts of ornithine did not negatively impact arginine biosynthesis itself or the production of polyamines from arginine by ADC, neither by limiting the availability of its substrate arginine nor *via* feedback inhibition of ADC by excess putrescine. Furthermore, arginine levels were not altered differently in Arabidopsis arginase overexpressing and arginase insertion mutant lines (Shi et al., 2013), pointing toward multiple mechanisms regulating arginine and polyamine biosynthesis.

Arginase T-DNA insertion mutants of Arabidopsis showed much higher expression levels of polyamine biosynthesis enzymes (*ADC1* and *ADC2*, *AIH*, *NLP1*) as well as higher putrescine and spermine contents, whereas arginase overexpressing lines had significantly lower mRNA levels of the analyzed enzymes compared to the wild type (Shi et al., 2013). This is consistent with another report demonstrating that reduced arginase activity in Arabidopsis transgenic lines led to a significant increase in putrescine concentration (Brauc et al., 2012).

Polyamines were found to be non-competitive inhibitors for δOAT (Stránská et al., 2010) in *Pisum sativum*. It seems that increased polyamine concentrations can significantly reduce the activity of pea δOAT *in vivo* and Stránská et al. (2010) hypothesized that this would result in slowing down arginine catabolism. Since polyamines are involved in diverse physiological responses, it could be advantageous for plants to slow down arginine catabolism in favor of polyamine synthesis if necessary.

### NO Production Involving Arginine

As a neutral and lipophilic gaseous molecule of small dimensions, NO can easily cross membranes, diffuse into the cytosol and bind to its soluble targets to act as a multifunctional signaling molecule. Beside implication of NO in plant growth and development from germination to fruit ripening and flowering, it is also generated in response to a wide range of biotic stresses, such as biotrophic and necrotrophic pathogens, as well as to a number of abiotic stresses, such as heavy metal, drought and salt stress (Neill et al., 2008; Moreau et al., 2010; Yu et al., 2014; Zhang et al., 2014; Domingos et al., 2015).

In plants, there are several sources of NO, including arginine and nitrite-dependent pathways, as well as non-enzymatic NO generation (Leitner et al., 2009; Froehlich and Durner, 2011). The role of arginine as a precursor for NO is increasingly apparent, although the molecular mechanism of NO production from arginine remains elusive. Biochemical assays and the effects of inhibitors of animal NO synthase (NOS) support the existence of NOS-like enzymes in plants, converting arginine into NO and citrulline (Zhang et al., 2014; Domingos et al., 2015). However, all attempts to identify such enzymes in higher plants failed so far, whereas a NOS protein was described in the green alga *Ostreococcus tauri* (Foresi et al., 2010). Based on enzymatic assays or staining techniques, NOS-like activity in plants has been associated with mitochondria, chloroplasts and peroxisomes (Froehlich and Durner, 2011).

Flores et al. (2008) proposed that mitochondrial arginase activity competes with arginine-dependent NO production in Arabidopsis, while Shi et al. (2013) showed that both Arabidopsis arginase isoenzymes are able to negatively regulate polyamine synthesis as well as NO synthesis. Leaving the existence of a NOS-like activity in plants speculative, other researches showed that polyamines could induce rapid biosynthesis of NO in root tips and primary leaves of Arabidopsis seedlings (Tun et al., 2006; Siddiqui et al., 2011; Wimalasekera et al., 2011; Tiburcio et al., 2014).

Both polyamines and NO function in regulation of plant development and as signaling molecules mediating a range of responses to biotic and abiotic stresses (Zhao et al., 2007; Neill et al., 2008; Lozano-Juste and León, 2010; Gupta et al., 2011; Wang et al., 2011). Further research is needed to decipher how arginine, arginine degradation and arginine-derived NO and polyamines influence each other to orchestrate development and plant defense response to stress.

## Conclusion

The present review gives an update on the recent advances in research on arginine metabolism in higher plants, mainly derived from work with Arabidopsis. Experimental evidence indicated that both pathways for ornithine biosynthesis, the well characterized cyclic pathway and the linear pathway using NAOD, are present in plants. Arginine levels in plant tissues seem to be regulated by a multitude of mechanisms, since most of the experimental manipulations of arginine biosynthesis or catabolism did not alter arginine concentrations. The role of arginine as important amino acid for nitrogen storage in plants is complemented by arginine catabolism mobilizing stored nitrogen and fine-tuning the production of NO, polyamines and potentially proline. While several regulatory mechanisms of arginine biosynthesis were identified, understanding of the regulation of the different pathways for arginine utilization requires further research.

Detailed biochemical and physiological characterization of the enzymes mediating arginine metabolism and the regulatory mechanisms allocating arginine-derived nitrogen to signaling, growth, reproduction or defense to stress will provide a better understanding of the role of arginine metabolism in nitrogen use efficiency in plants. Knowledge about essential intermediates and developmental switches between nitrogen storage and re-mobilization may help to improve crop plants or cultivation conditions. Optimized nitrogen use efficiency of crop plants will be crucial to reduce detrimental effects of nitrogen shortage on productivity and will help to avoid negative economic and environmental impact of excessive nitrogen fertilization in agriculture.

## Author Contributions

All authors have contributed significantly and approved the final manuscript.

### Conflict of Interest Statement

The authors declare that the research was conducted in the absence of any commercial or financial relationships that could be construed as a potential conflict of interest.
